# Increased Stress Resistance and Lifespan in *Chaenorhabditis elegans* Wildtype and Knockout Mutants—Implications for Depression Treatment by Medicinal Herbs

**DOI:** 10.3390/molecules26071827

**Published:** 2021-03-24

**Authors:** Janine Naß, Christopher J. Kampf, Thomas Efferth

**Affiliations:** 1 Department of Pharmaceutical Biology, Institute of Pharmaceutical and Biomedical Sciences, Johannes Gutenberg University, Staudinger Weg 5, 55128 Mainz, Germany; janine.nass@uni-mainz.de; 2 Department for Chemistry, Johannes Gutenberg University Mainz, Duesbergweg 10-14, 55128 Mainz, Germany; kampfc@uni-mainz.de

**Keywords:** ageing, *C. elegans*, depression, phytochemical, phytotherapy, stress

## Abstract

Depression and anxiety disorders are widespread diseases, and they belong to the leading causes of disability and greatest burdens on healthcare systems worldwide. It is expected that the numbers will dramatically rise during the COVID-19 pandemic. Established medications are not sufficient to adequately treat depression and are not available for everyone. Plants from traditional medicine may be promising alternatives to treat depressive symptoms. The model organism *Chaenorhabditis elegans* was used to assess the stress reducing effects of methanol/dichlormethane extracts from plants used in traditional medicine. After initial screening for antioxidant activity, nine extracts were selected for in vivo testing in oxidative stress, heat stress, and osmotic stress assays. Additionally, anti-aging properties were evaluated in lifespan assay. The extracts from *Acanthopanax senticosus, Campsis grandiflora, Centella asiatica, Corydalis yanhusuo, Dan Zhi, Houttuynia cordata, Psoralea corylifolia, Valeriana officinalis*, and *Withania* *somnifera* showed antioxidant activity of more than 15 Trolox equivalents per mg extract. The extracts significantly lowered ROS in mutants, increased resistance to heat stress and osmotic stress, and the extended lifespan of the nematodes. The plant extracts tested showed promising results in increasing stress resistance in the nematode model. Further analyses are needed, in order to unravel underlying mechanisms and transfer results to humans.

## 1. Introduction

Depression and anxiety disorders are widespread diseases. They affect mood, sleep, joy of life, and the will to live. Over 264 million people suffered from depression in 2017, according to numbers from the World Health Organization [1]. Depression is a leading cause of disability and it is one of the greatest burdens on healthcare systems worldwide. The COVID-19 pandemic represents an unprecedented global challenge. In addition to the direct problems for the health systems and the people affected, there is a growing number of problems that are the consequence of COVID-19 measures. Isolation, fear of losing one’s job, threats to one’s life, as well as fewer social contacts are factors that contribute to the dramatic increase in the number of depressions and anxiety disorders during pandemic [2,3].

Health care systems all over the world can hardly cope with these increasing patient numbers [4]. Countries, in which the health system is not well developed and where people live in poor condition and are especially hard hit by the pandemic, are particularly affected. People in poor countries hardly have access to medication for the treatment of depression [5], and, secondly, they are hit particularly hard by the pandemic [6,7,8], which implies that the number of patients suffering from depression will rise. In poor regions, the number of people suffering from trauma and depression has increased [9,10,11], which further enhances the risk of depression. A study that examined the consequences of the pandemic in a township in South Africa showed that people, who are pre-burdened by childhood trauma, were at greater risk of developing depression during quarantine [12]. According to the WHO, a study in Amhara Regional State, Ethiopia, a three-fold increase in the prevalence of depression was recorded [13]. These figures illustrate an emerging need for antidepressant therapies in these countries.

As of yet, the exact molecular pathomechanisms of depression are still unclear. Depression is a multifactorial disease that results from the interplay between many genetic [14,15] and non-genetic factors [16,17].

The studies focused on both the serotonin [18] and the dopamine system [19]. This is reflected by established conventional therapies, which mainly consist of selective serotonin reuptake inhibitors (SSRIs), monoaminoxidase (MAO) inhibitors, and tricyclic antidepressants (TCA) [20].

One of the strongest risk factors for depression and mental illness is exposure to chronic stress. Chronic stress can lead to behavioral and genetic changes [21]. Beside psychological stressors, oxidative stress seems to play a pivotal role. In some studies, oxidative stress marker levels differed in depressed patients from those in healthy subjects. Oxidative stress leads to DNA damage, protein damage, and lipid peroxidation [22], which ultimately contribute to concomitant diseases, such as cardiovascular diseases [23]. Therefore, it can be postulated that antidepressant therapies have to fulfil several functions, i.e., lowering oxidative stress and reactive oxygen species (ROS) generation and increasing levels of neurotransmitters, such as serotonin and dopamine.

In recent years, the treatment options for depression did not considerably change and they are still unsatisfactory [24]. Although there are some drugs for anti-depressive treatment, their success rate is limited, partly because of severe side effects [25].

In developing countries with weak health systems, the supply of expensive drugs may be also restricted. Medicinal plants represent effective alternatives in this context [26,27]. They have been used in traditional medicine for long times and they are frequently well tolerated [28]. They are affordable to people with low income and are indispensable for primary health care in the Third World [29]. Herbal medicines act on the nervous system and they show antidepressant effects by influencing the levels of serotonin, dopamine, or norepinephrine [30,31]. In addition, many of them show anti-inflammatory and radical scavenging properties [32,33]. For example, St. John’s wort is a well-known herbal drug, which is widely distributed for the treatment of mild to moderate depression [34,35].

In the present study, we investigated the potential of medicinal plants that are traditionally used against depression and to evaluate their stress-reducing and antidepressant properties in *Caenorhabditis elegans*. *C. elegans* is a widely used model in experimental pharmacology for investigating substances for their anti-stress and antidepressant properties [36,37,38,39,40]. The experimental results can be well transferred due to the high similarity of the signaling pathways between humans and *C. elegans*. Mutant *C. elegans* strains with deficiencies in the serotonin and dopamine system, as well as with deficiencies in the oxidative system, were selected to imitate a depressive state, in order to elucidate the contribution of neurotransmitters and oxidative stress to depressive symptoms.

**Figure 1:**
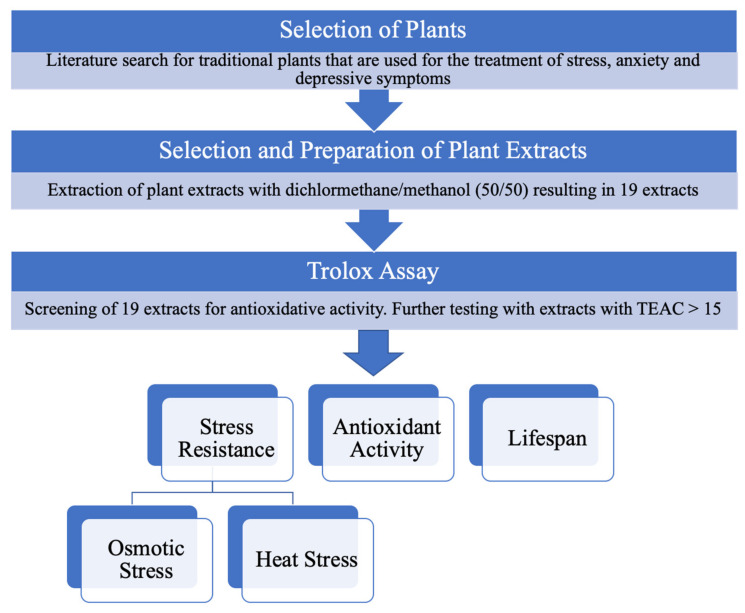
Schematic diagram of the process of selection of plants and experiments carried out.

Based on a literature search, we identified 19 medicinal plants that are traditionally used to treat depressive moods and stress. These 19 plants were tested for their antioxidant and stress reducing properties (Figure 1; Table 1).

## 2. Materials and Methods

### 2.1. Selection of Plants

We searched for medicinal plants that are traditionally used to treat stress symptoms and anxiety using the PubMed database. In addition to plants of traditional Chinese and Indian medicine, plants from traditional European medicine that have already been proven to be effective in the treatment of anxiety and depression were included in the present investigation (see Table 1).

*Valeriana officinalis* L, *Humulus lupulus* L, and *Passiflora incarnata* L. are traditionally used in Europe to treat sleep disorders and mild depression. These applications are also reported by the Committee on Herbal Medicinal Products (HMPC) of the European Medicines Agency’s (EMA). HMPC concludes that the use of these plants is well-established, safe, and effective. The other plants selected are used in Traditional Chinese Medicine to treat depressive symptoms. Additionally, most of them are used to treat inflammation (Table 1).

### 2.2. Substances and Reagents

All of the reagents were purchased from Merck KgA Darmstadt, if not stated otherwise. Dichloromethane and methanol were of high analytical grade. Standardized plant material in pharmaceutical quality was ordered from certified trader (HerbaSinica Hilsdorf GmbH, Rednitzhembach, Germany) and stored at concentrations of 100 mg/mL. The extract dilutions were performed with M9 buffer (according to standard protocols) to a final concentration of 100 µg/mL with 0.1% DMSO. Purified deionized water was prepared using the Milli Q Water Purification system from Millipore (Millipore Corp., Bedford, MA, USA). 96-well polypropylene flat-bottomed microplates were purchased from Greiner Bio-One GmbH (Frickenhausen, Germany). For *C. elegans* cultivation and assays, Petri dishes (60 mm or 35 mm diameter) were obtained from Sarstedt (Nümbrecht, Germany) as well as conical tubes 15 and 50 mL.

### 2.3. Plant Material and Extraction Preparation and Reference Compounds

The plant material was purchased from certified traders (HerbaSinica and Alfred Galke GmbH, Bad Grund, Germany). Each plant material was analyzed for identity and purity, heavy metals, plant treatment products, and microbiology according to the applicable pharmacopoeias, i.e., European Pharmacopoeia and Chinese Pharmacopoeia. The results and release were approved by an expert person according to German Medicines Act §65.

Each 100 g plant material was dried and milled. Extraction was performed as cold maceration with dichloromethane and methanol (50:50 *v*/*v*) 250 mL per 100 g plant material. The stoppered flasks were gently shaken overnight at room temperature. To remove plant material, the macerate was filtered through Whatman no.1 filter paper (Merck KGaA, Darmstadt, Germany). The evaporation of extracts was performed under reduced pressure with rotatory evaporator at 40–50 °C. All of the extracts were dissolved in dimethylsulfoxide (DMSO) at a concentration of 100 mg/mL (stock solution) and stored at −20 °C until use. Each stock solution was diluted in the desired medium to reach 100 µg/mL before use.

The reference compounds were ursolic acid (≥95%, Sigma Aldrich; Taufkirchen, Germany), withanolide A (>95%; Cayman Chemical; Ann Arbor, MI, USA), and withanone (Phyproof^®^ reference substance; Sigma Aldrich).

### 2.4. C. elegans Strains and Culture Conditions

The study design was adopted from our previous studies [41,42,43]. The data of untreated controls (osmotic stress and lifespan) were taken from these studies for comparison, since the experiments were partially run in parallel. Different *C. elegans* mutants were selected for the in vivo experiments. The selected mutants have deficiencies in proteins that affect the serotonin system, the dopamine system, and antioxidant enzymes (Table 2). *C. elegans* strains (Bristol N2, DA1814 (*ser-1*), AQ866 (*ser-4*), DA2100 (*ser-7*), DA2109 *(ser-1/ser-7*)*, MT9772* (*mod5*), RB665 (*dop-1*), LX702 (*dop-3*), VC289 (*prdx2*), and QV225 (*skn1*), as well as *Escherichia coli* OP50, were obtained from the Caenorhabditis Genetics Center (CGC, University of Minnesota, USA), which is funded by the National Institutes of Health Office of Research Infrastructure Programs (program no. P40 OD010440). The worms were cultured at 20 °C on nematode growth medium (NGM) that was prepared according to standard protocol with 100 µL *E. coli* OP50 standard solution as the food source, as previously described [44].

The protocol for synchronization of worms was provided by Prof. Dr. med. Hammes (University Mannheim, Mannheim, Germany). Worms are allowed to lay eggs for approximately two days. After these days, the worms are washed of plates with 2 mL M9 buffer/plate and pelletized in 15 mL tubes (2 min, 1800 rpm). The supernatant is discarded down to 0.5 mL and the pellet is resuspended in 8 mL bleaching solution consisting of 2 mL sodium hypochlorite solution 12% (Carl Roth, Karlsruhe, Germany), 16 mL autoclaved purified water and 6 mL 2M NaOH 98% (Carl Roth, Karlsruhe, Germany). The worms were bleached for about 10 min. (overhead mixing and every 2–3 min. for 20 s of vigorous pre-writing). After worms have been dissolved, eggs were washed twice with 10 mL M9 buffer. Tubes are centrifuged (2 min, 1800 rpm) to remove supernatant and pellet is resuspended in M9 buffer through vigorous vortexing. After washing, the resuspended pellet is dissolved in 1 mL M9 buffer and plate out about 150 µL eggs/plate. Age-synchronized L4 worms were obtained three days after synchronization.

NGM plates for assays were supplemented with the extracts stock solution by insertion into the autoclaved NGM at 50 °C.

### 2.5. Trolox Antioxidant Activity

The Trolox equivalent antioxidant capacity assay was used to compare the antioxidant activity of extracts towards the antioxidant effect of ((±)-6-hydroxy-2,5,7,8-tetramethylchromane-2-carboxylic acid (Trolox, Sigma-Aldrich). A radical cationic solution was used to measure the absorbance differences. Antioxidant extracts would inhibit the absorbance. ABTS**^·^**^+^ (2,2-azinobis-(3-ethylbenzothiazoline-6-sulfonate, Sigma Aldrich) was generated by oxidation while using potassium persulfate for at least 12 h. EtOH 96% (Merck KGaA, Darmstadt, Germany) was used to dilute the solution to an absorbance of 734 nm.

Trolox and extracts were diluted to concentrations that were between 0.1 mM and 0.00625 mM with 96% EtoH. For each run, solvent blank and positive controls with ABTS^·+^ solution were performed. The absorbance was measured after 6 min. with an Infinite M2000 Pro™ plate reader (Tecan, Crailsheim, Germany). The decreased absorbance that was caused by Trolox was plotted for the different concentrations tested. The resulting plot was used as a calibration curve. The slope of the absorbance inhibition vs. antioxidant concentration plot was divided by the slope of the Trolox plot to calculate the Trolox equivalent antioxidant capacity (TEAC) as Trolox equivalents (TE) in mM/mg plant extract.

### 2.6. H2DCFDA Antioxidant Activity

2,7-Dichlorodihydrofluorescein diacetate (H_2_DCFDA; Sigma-Aldrich), a cell-permeable fluorogenic substance, was taken to analyze the amount of reactive oxygen species (ROS) in *C. elegans* and the influence of extracts on ROS levels according to Yoon et al., 2018 [45], with minor modifications. H_2_DCF is oxidized by ROS, leading to the formation of the fluorescent dye dichlorofluorescein (DCF). The fluorescence intensity is proportional to the ROS levels in *C. elegans*.

Age-synchronized L4 larvae were treated with 100 µg/mL of each extract or DMSO 0.1% (control) for 24 h. Afterwards, 50 nematodes per group were collected in a tube using 100 µL phosphate-buffered saline (PBS, Sigma-Aldrich) with 1% Tween-20 (Sigma-Aldrich). Nematodes were sonicated to obtain the worm lysate. The lysates were then transferred to 96-well microtiter plates. The samples were incubated with 50 µM H_2_DCF-DA in PBS. After 2 h, intracellular ROS concentrations was measured at an extinction wavelength of 485 nm and an emission wavelength of 530 nm with an Infinite M2000 Pro™ plate reader (Tecan). The treated and untreated nematodes were compared for their ROS levels/fluorescence intensity.

### 2.7. Osmotic Stress Resistance Assay

A total of 100 age-synchronized L1 larvae were treated with 100 µg/mL of each extract or 0.1% DMSO as a control for 72 h, respectively. After three days, *C. elegans* were placed on NGM plates containing 500 mM NaCl (≥99.5%; Carl Roth) at 20 °C. The worms were scored for movement every 2 min, until all worms were paralyzed [46].

### 2.8. Heat Stress Assay

The heat stress assay was performed as described [47]. A total of 100 age-synchronized worms were bred on NGM agar containing either 100 µg/mL extract or 0.1% DMSO as control. L4 larvae were transferred to fresh NGM plates and then incubated at 37 °C for 6 h. After heat shock plates were returned to 20 °C overnight. Worms were scored for survival after 20 h. The worms that did not move and respond to a stimulus with a platinum wire were counted as dead.

### 2.9. Lifespan Assay

Lifespan assays were performed with age-synchronized L4 larvae *C. elegans* at 20 °C. Lifespan assay NGM plates contained ampicillin (Amp) and floxuridine (FUDR) to inhibit egg laying and contamination with foreign bacteria. Therefore, 33 µL of 150 mM FUDR (Merck KGaA) and 100 µL of 100 mg/mL Ampicillin (anhydrous 96–102%, Sigma–Aldrich) per 100 mL NGM were added to 55 °C NGM [48].

Plates either contained 100 µg/mL of plant extract or 0.1% DMSO as the control. One-hundred worms were used for each strain or condition being tested. Survival was scored every 24 h. Worms were tested for survival with a platinum wire. Worms were considered to be dead if they did not respond upon prodding with the tip.

### 2.10. Extract Analyses by HPLC-HRMS

The following method has been applied with the same equipment and similar settings, as previously reported by us [49]. Dried extracts were dissolved in MeOH at a concentration of 2 mg/mL and diluted by a factor of 1000. Analysis was accomplished using a 1260 Infinity II high-performance liquid chromatography (HPLC) system (Agilent Technologies, Waldbronn, Germany) coupled to a 6545 QTOF mass spectrometer (Agilent Technologies) coupled to an Agilent Jet Stream electrospray ionization (ESI) interface. For chromatographic analysis, an EclipsePlus C18 RRHP (50 mm × 2.1 mm, 1.8 μm, Agilent Technologies) column was selected. The column was eluted with the following gradient consisting of 2% acetonitrile in H2O (A) and 2% H2O 0.1% HFo in acetonitril (B): starting at 2% B, ramping to 70% B in 10 min, further ramping to 95% B at min. 20, staying at 95% B for 10 min, going back to 2% B in 2 min, and re-equilibrating the column at 2% B for 8 min. before the next run.

The flow rate was set to 0.2 mL/min. The injection volume of the samples was 2 μL per injection. Analyses were performed in positive and negative ion automated data-dependent acquisition mode. Full mass spectrometry (MS) scans from m/z 100–1500 Da were gained at a scan rate: of 1 spectrum/s. The conditions for ESI were chosen, as follows: capillary voltage 3.5 kV, nozzle voltage 1 kV, fragment 175 (arbitrary units), drying gas temperature 320 °C, sheath gas temperature 300 °C, drying gas flow 8 L/min, and nebulizer pressure 25 psig. Nitrogen was used as the nebulizer and auxiliary gas. The data were attained in centroid mode. The instruments were calibrated right before each sample set.

### 2.11. Statistical Analysis

All of the experiments were performed in triplicate. Trolox assay results are represented as mean ± standard deviation. Data from lifespan and osmotic stress resistance assay were analyzed using Kaplan–Meier analysis and log-rank (Mantel Cox) test followed by Gehan-Breslow-Wilcoxon test. The results are represented as mean ± standard error of mean (SEM). The significance of difference between control and treated groups was analyzed by one-way analysis of variance (ANOVA) followed by Bonferroni’s method. *p*-values < 0.05 were accepted as statistically significant. Graphs were built with GraphPad Prism version 5.00 for Windows (GraphPad Software, San Diego, La Jolla, CA, USA) or Microsoft Excel (Microsoft Corporation (2018)).

## 3. Results

### 3.1. Trolox Antioxidant Activity

**Figure 2:**
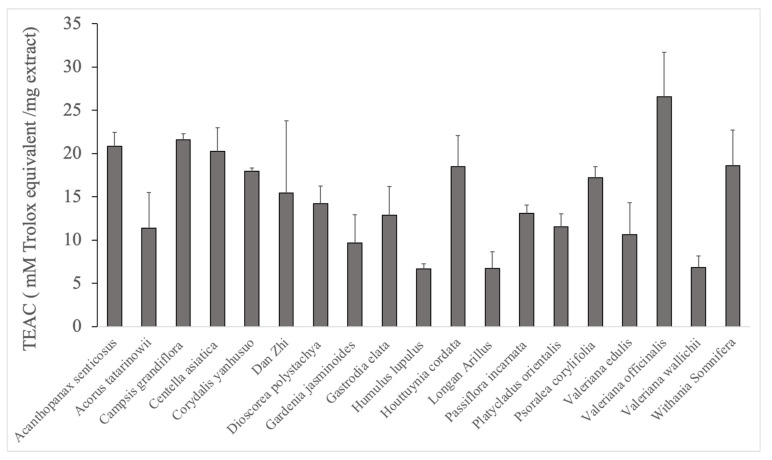
Antioxidant activity as determined by the TEAC assay. TEAC is represented as mean with standard deviation.

The Trolox Equivalent Antioxidant Capacity (TEAC) assay is a useful and widely used test to determine the antioxidant properties of substances [50]. Because antioxidant properties play an important role in the treatment of depression [51,52], this test was used as a first screening test (Figure 2). Only those extracts with a TEAC above 15 TE were selected for further analysis because many plant extracts are known to exert antioxidant effects. These were *Acanthopanax senticosus* (20.83 ± 1.6), *Campsis grandiflora* with a TEAC of 21.57 0.7, *Centella asiatica* (20.23 ± 2.7), *Corydalis yanhusuo* (17.92 ± 0,4), *Dan Zhi* (composition of this herbal mixture: see Table 1) (15.45 ± 8.3), *Houttuynia cordata* (18.51 ± 3.5), *Psoralea corylifolia* (17.19 ± 1.3), *Valeriana officinalis* (26.55 ± 5.1), and *Withania somnifera* (18.59 ± 4.1). The TEAC assay showed that nine of the 19 extracts revealed a TEAC of more than 15. Of those, *Valeriana officinalis* showed the highest TEAC.

### 3.2. H2DCFDA Antioxidant Activity

The cell-permeable H_2_DCFDA—also known as dichlorofluorescin diacetate—is a chemically reduced form of fluorescein that is widely used as an indicator of ROS in cells or *C. elegans*, to detect ROS generation [53]. It was used to test the antioxidant properties of the selected nine extracts in vivo. Therefore, wild-type and mutants *C. elegans* L4 larvae were treated with 100 µg/mL extract for 24 h. Subsequently, the intracellular ROS concentration was measured.

**Figure 3:**
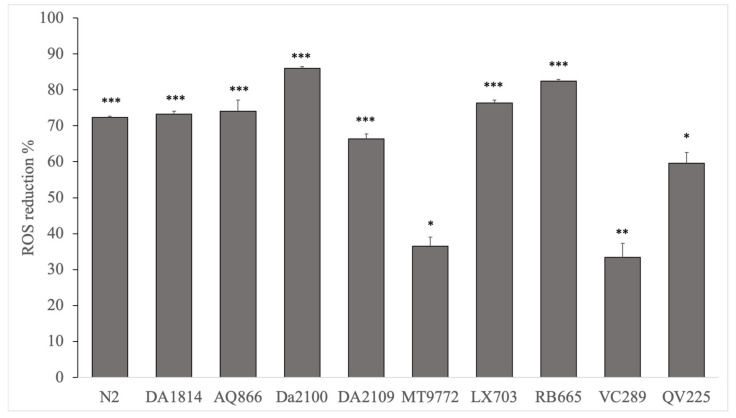
Antioxidant activity, as determined by the H_2_DCFDA assay for the extract *Acanthopanax senticosus*. *** *p* < 0.001, ** *p* < 0.01, and * *p* < 0.05.

The H_2_DCFDA assay showed a strong reduction of ROS in all *C. elegans* tested (Table 3). *Houttuynia cordata, Centella asiatica, Corydalis yanhusuo, Withania somnifera*, and *Acanthopanax senticosus* (Figure 3) showed particularly strong reductions in oxygen radicals.

For the wild type nematode N2, *Valeriana officinalis* showed the strongest antioxidant, activity with 81.4% ± 2.5 (*p* < 0.001), followed by the mixture *Dan Zhi* (80.2 ± 2.5, *p* < 0.001). *Psoralea corydalis* showed the lowest antioxidant activity (45.4% ± 2.0, *p* < 0.001). For mutant DA1814 (*ser-1*), *Acanthopanax senticosus* showed the strongest effect (73.3% ± 0.7, *p* < 0.001), followed by *Withania somnifera*, with 72.2% ± 3.9 (*p* < 0.001). Again, *Dan Zhi* is the extract with the lowest effect, with a 22% reduction in the radical oxygen species (*p* < 0.05).

In the mutant AQ866, *Corydalis yanhuso* was the most effective (81.5% ± 0.4, *p* < 0.001), whereas *Campsis grandiflora* showed no significant effect.

In DA2100 (*ser-7*), extracts of *Acanthopanax senticosus, Withania somnifera*, and *Corydalis yanhuso* were very active. They significantly reduced ROS (*p* < 0.001) by 80%.

DA2109 (*ser-1*/*ser-7*) showed a comparable result.

The serotonin transporter deficient mutant MT9772 showed the lowest ROS reductions in the experiments after treatment. *Centella asiatica* (67.7% ± 1.0, *p* < 0.001) and *Psoralea corydalis* (40.1% ± 14.1, *p* < 0.05) were most effective.

In the *dop-1*-deficient RB665 mutant, ROS was the best reduced by *Acanthopanax senticosus* (82.4% ± 0.5, *p* < 0.001). *Dan Zhi* (35.9% ± 6.2, *p* < 0.01) showed the least effect.

*Houttuynia cordata* reduced ROS of the *dop-3*-deficient mutant LX703 by 85% (*p* < 0.001). *Acanthopanax senticosus* and *Withania somnifera* also showed strong ROS reducing properties. *Valeriana officinalis* showed no significant effect.

The VC289 mutant, which has a mutation in the redox protein peroxiredoxin-2, has the strongest effect *Withania somnifera* (87.8% ± 9.7, *p* < 0.01). *Centella asiatica* had the least effect, with a ROS reduction of 28.3% ± 2.4 (*p* < 0.01).

*Psoralea corydalis* has the strongest effect on the mutant QV225 (*skn-1*). Here, the ROS levels were reduced by up to 78% (*p* < 0.01). *Dan Zhi* showed the lowest effect here, although the radicals were still reduced by approximately 50%.

### 3.3. Acute Osmotic Stress Resistance Assay

The acute osmotic stress resistance assay is an experiment that shows how nematodes can resist acute changes in osmotic concentrations that are indicated by time in minutes, until worms are paralyzed. This test is widely used to test the stress resistance of organisms [54].

The osmotic stress assay showed that the plant extracts prolonged nematode survival under hyperosmotic conditions (Table 4). Among the control nematodes, the wild type had the longest survival time, with 2.6 ± 0.3 min. The mutants lived shorter on average.

For the wild-type, *Campsis grandiflora* and *Valeriana officinalis* prolonged survival the most (284.6% ± 8.1, *p* < 0.001 and 296.2% ± 5.2 *p* < 0.001). *Acanthopanax senticosus* showed the least effect, with 153.8% ± 7.5 (*p* < 0.05).

For the *ser-4* deficient mutant AQ866, *Campsis grandiflora* and *Centella asiatica* showed the longest survival with 277.3% ± 8.2 (*p* < 0.001) and 295.5% ± 7.7 (*p* < 0.001). Thus, the two extracts prolonged survival by approximately 4 min. on average when compared to the control. *Acanthopanax senticosus* showed the lowest survival extension.

For the DA1814 mutant, survival was increased from 2 min. (control)—to 7.14 ± 1.24 min. by *Withania somnifera* (338.1% ± 16.9; *p* < 0.001). *Valeriana officinalis* extended survival to 6.9 ± 0.43 min. (328.6% ± 5.8; *p* < 0.001).

In mutant DA2100 animals, the survival of *Dan Zhi* treated animals was increased to 363.6% ± 3.8 (*p* < 0.001) as compared to untreated control.

The *ser-1*/*ser-7* deficient mutant DA2109 lived longest (243.5% ± 10.7, *p* < 0.001) if treated with *Valeriana officinalis*.

For the serotonin transporter-deficient mutant MT9772, *Campsis grandiflora* showed the best results. Here, survival is extended by 3 min. on average (*p* < 0.001). *Acanthopanax senticosus* showed no effect compared to the control (91.7% ± 9.1; n.s.).

In the dopamine-deficient mutant RB665, *Centella asiatica* (310.0% ± 8.19, *p* < 0.001) and *Acanthopanax senticosus* (395.0% ± 11.4, *p* < 0.001) revealed the best effects. Here, survival was extended by 4 and 5.5 min, respectively.

For the dopamine D3 deficient mutant LX703, *Dan Zhi* showed the best result (271.4% ± 7.0, *p* < 0.001), followed by *Withania somnifera* (139.1% ± 37.7, *p* < 0.05).

The VC289 strain (*prdx-2-*deficient) showed an extended survival of 4.7 min. (± 0.5, *p* < 0.01) upon treatment with *Valeriana officinalis*. In contrast, the QV225 worms benefitted most from the treatment with *Campsis grandiflora* (235.0% ± 8.5, *p* < 0.001). Overall, *Acanthopanax senticosus* showed the smallest increase in survival time. *Valeriana officinalis* showed the best overall lifespan extension (232.8% ± 48.5).

### 3.4. Heat Stress Assay

The heat stress assay is a well-established experiment that is used to test the tolerance towards higher temperatures upon drug treatment [55]. Increasing stress resistance is expressed as percentages of higher survival rates of worms.

**Figure 4:**
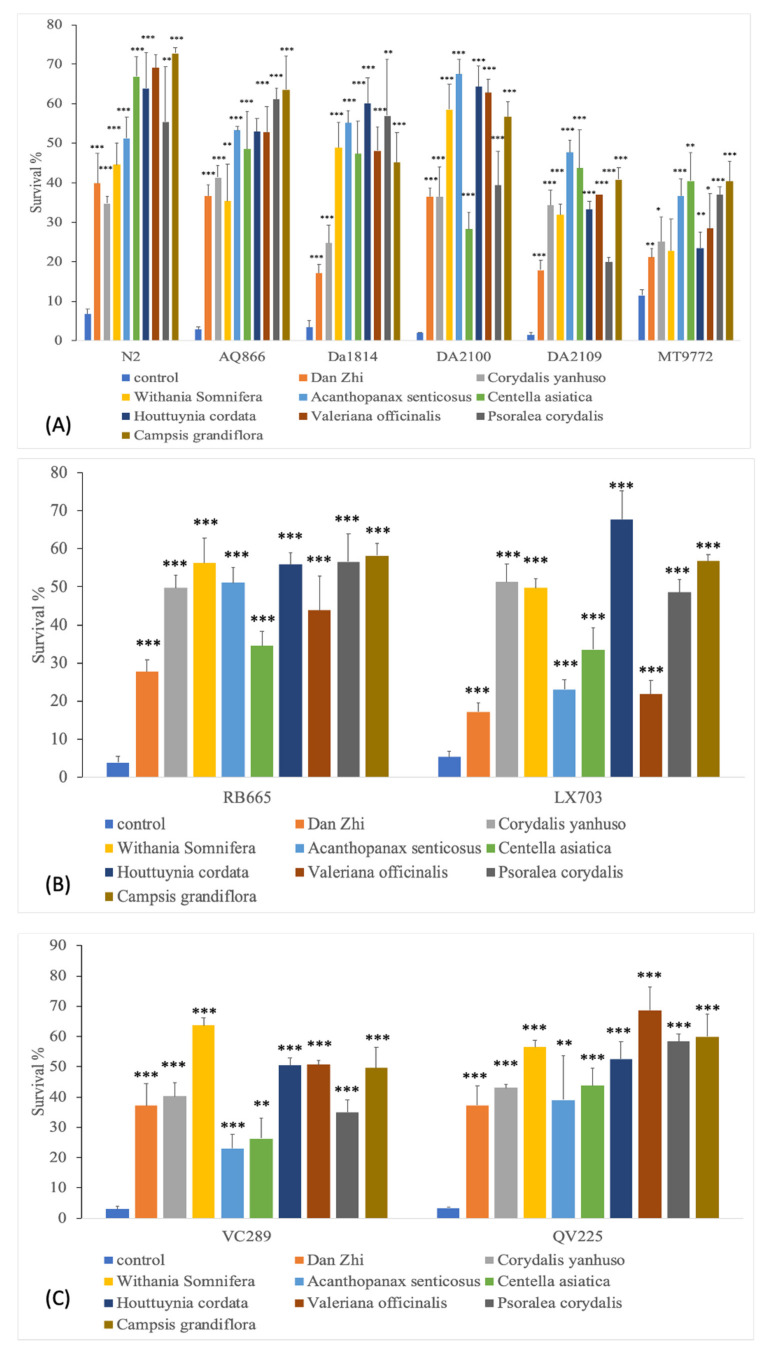
Effect of plant extracts on heat stress. (**A**) Serotonin-deficient mutants. (**B**) Dopamine-deficient mutants. (**C**) ROS mutants. *** *p* < 0.001, ** *p* < 0.01 and * *p* < 0.05.

In the overall view, all of the tested substances increased the number of surviving worms compared to the untreated controls. *Dan Zhi, Corydalis yanhuso*, and *Centella asiatica* revealed significant, but lower, effects than the other extracts. Figure 4 shows an overview of results the survival of serotonin, dopamine, and ROS mutants.

The N2 wild-type strain showed a survival of 7% in the untreated control. Treatment with the plant extracts increased the number of surviving animals up to 73% (*Campsis grandiflora p* < 0.001). The weakest effect is observed with *Dan Zhi*, with 40% (*p* < 0.001).

*Psoralea corydalis* and *Campsis grandiflora* increased the survival to 62% (*p* < 0.001) in AQ866 mutant worms. In DA1814 (*ser-1-*deficient) worms, *Houttuynia cordata* achieved the best effect, with 60% (*p* < 0.001). For the DA2100 (*ser-7*) and DA2109 (*ser-1/ser-7*) strains, *Acanthopanax senticosus* was most effective (67.5% and 47.7%, respectively, *p* < 0.001). The MT9772 (*mod-5*) mutants showed a 25% increase in survival after treatment with *Psoralea corydalis* (*p* < 0.05). *Campsis grandiflora* increased the survival from an average of 3.8% to 58% in the dopamine-deficient RB665 mutant. *Houttuynia cordata* was most effective (68%, *p* < 0.001), followed by *Campsis grandiflora* (57%, *p* < 0.001) in LX703 worms. In the ROS-deficient mutants VC289 (*prdx-2*) and QV225 (*skn-1*), *Withania somnifera* and *Valeriana officinalis* were most effective (64% and 69%, *p* < 0.01). Overall, *Campsis grandiflora* showed the highest heat stress resistance induction (54.4% *±* 4.8).

### 3.5. Lifespan Assay

Because genetic modifications can considerably influence the survival of *C. elegans*, studies on the survival and aging of the nematodes have become an important feature for the research of effective substances [56]. Therefore, the lifespan of the different nematode strains was investigated. Table 5 shows an overview of the average lifetime after treatment with the different extracts.

All of the extracts were able to prolong the life of the worms.

The deficient mutants generally showed a shortened lifespan when compared to the wild type. Untreated wildtype N2 animals showed a survival of 19.7 ± 0.2 days. Treatment with *Campsis grandiflora* prolonged the lifespan up to 23.2 ± 0.7 (117.7% ± 3.02; *p* < 0.001). *Dan Zhi* prolonged lifespan to 23.2 ± 0.6 days (*p* < 0.001).

Treatment with *Centella asiatica* extended the lifespan of the *ser-4* mutant AQ866to 23 days in mean (*p* < 0.001). The lowest effect is observed for *Campsis grandiflora*, which extended lifespan to 19 days (125.3% ±3.19; *p* < 0.001).

*Dan Zhi* extended lifespan of the DA1814 mutant to 131.8% ± 2.05 (24.4 ± 0.5 days; *p* < 0.001). *Valeriana officinalis* prolonged the lifespan to 24 days (*p* < 0.001).

The DA2100 (*ser-1-*deficient) lifespan was extended to 137.7% ± 1.35 upon treatment with *Dan Zhi*. In sum, all of the extracts prolong the lifespan in DA2100 between 20 and 22 days.

For DA2109 (*ser-1/ser-7*) *Valeriana officinalis* extended lifespan to 22.3 ± 0.3 days (133.7% ± 1.38). The other extracts showed similar lifespan extensions (between 120 and 131%).

MT9772 nematodes showed a lifespan of 152.8% ± 2.01 upon *Valeriana officinalis* treatment when compared to the untreated control (*p* < 0.05).

The dopamine-deficient RB665 mutant (*dop-1*) revealed a prolongation in survival upon treatment with *Campsis grandiflora* to 157.7% ± 2.03 (*p* < 0.001).

The *dop-3-*deficient LX703 mutant showed a similar lifespan when compared to RB665. *Corydalis yanhuso* extended the lifespan to 22.4 ± 0.11 (*p* < 0.05). In total, lifespan was prolonged by all extracts. The smallest effect was observed upon treatment with *Centella asiatica* (114.9% ± 3.24, *p* < 0.001).

For the ROS-deficient mutant VC289, the mean lifespan was extended to 21 days by *Withania somnifera* (*p* < 0.05).

The mutant QV225 showed a mean extension of nine days after treatment with *Acanthopanax senticosus* (*p* < 0.001).

### 3.6. Extract Analyses by HPLC-HRMS

The extracts were analyzed for their main constituents while using high-performance liquid chromatography-electrospray ionization (HPLC-ESI) system. Chromatograms of positive and negative ion mode are provided in the Supplementary Materials Tables, which show the main peaks and retention time. The most intense peaks were evaluated for their identity using literature and databases, such as Massbank (https://massbank.eu/MassBank/; date accessed: 6 March 21), Chemspider (http://www.chemspider.com/; accessed on 6 March 2021) and Pubchem (https://pubchem.ncbi.nlm.nih.gov/; accessed on 6 March 2021). The identification of the most likely molecules was provided where possible. Additionally, extracts were evaluated for the presence of ursolic acid, withanolide A, and withanone when compared to reference substances.

The analysis of extracts revealed a high number of secondary metabolites, such as flavonoids and fatty acids. The following ingredients were detected in the extracts: Ursolic acid was present in the extracts of *Acanthopanax senticosus, Campsis grandiflora, Centella asiatica, Corydalis yanhusuo, Dan Zhi, Houttuynia cordata, Valeriana officinalis*, and *Withania somnifera.* Withanolide A and its stereoisomer withanone were found in the *Withania somnifera* extract as well as in *Centella asiatica* (see Supplementary Materials Files).

Additionally, luteolin [57] and maslinic acid [58] were identified in *Campsis grandiflora*. Madecassic acid [59] was identified in *Centella asiatica*. The extract of *Corydalis yanhusuo* contained the alkaloid panicudine. *Dan Zhi* is a Chinese herbal formulation containing nine different plants. The extract revealed pleionesin C and myosmine. *Houttuynia cordata* contained linolenic acid [60]. Additionally, quercetin and isomoreollic acid were identified. Wighteone [61], sophoracoumestan A [62], and bavachinin [61] were found in *Psoralea corylifolia*. Linoleic acid [63] was identified in *Withania somnifera*.

## 4. Discussion

Numerous plants are used in traditional medicine to treat depressive moods, anxiety, and sleep problems. Our investigation showed that the selected methanol/dichloromethane plants extracts have antioxidant properties in vitro and in vivo. Thus, our results are in line with other studies reporting on the antioxidant properties of medicinal plants [64,65].

Various solvents of differing polarities have to be used in order to extract different phenolic compounds from plants with accuracy [66]. Therefore, we used methanol as polar solvent and dichlormethane as unipolar solvent. As the preparation of extracts includes the removal of organic solvents obtaining a solvent free extract, there are many options for practical application, e.g., extracts could be re-dissolved in water or could be applied as liquid or dry extracts filled into capsules.

All of the extracts revealed more antioxidative activity than the reference substance, Trolox. The 9 selected plants—Acanthopanax senticosus, Campsis grandiflora, Centella asiatica, Corydalis yanhusuo, Dan Zhi, Houttuynia cordata, Psoralea corylifolia, Valeriana officinalis and Withania somnifera—all showed TEAC values above 15. Our results are supported by other authors. For example, studies on Acanthopanax senticosus confirmed that the plant is rich in polyphenols and it has antioxidant activity [67].

In addition to the Trolox assay, we also investigated the antioxidant properties of the nine selected substances in vivo. For this purpose, the *C. elegans* wild type and various mutants were used. Serotonin-deficient mutants, dopamine deficient-mutants and mutants with deficiencies in the antioxidant protein Prdx2 and the transcription factor Skn1 were used. Because serotonergic neurotransmission is associated with depression, different serotonin-deficient mutants were selected to represent this clinical picture. A lack of serotonin resulted in increased vulnerability to stress [68]. The same applies to dopamine. A lack of dopamine was associated with depression [69]). In recent years, an increasing number of studies reported on the connection between ROS and depression. Preclinical studies demonstrated that the transcription factor Nrf2 was downregulated in a murine model of depression [70]. In *C. elegans*, the detoxification processes were initiated by Skn1 [71]. Additionally, *Skn-1* was activated through PMK-1/p38 MAP-kinase-dependent phosphorylation upon oxidative stress [72]. For this reason, the mutant QV225 (*skn-1*) was included in our experiments. *Skn-1* is the *C. elegans* orthologue of human *Nrf2*. Prdx2 belongs to the peroxiredoxin family of antioxidant ROS-scavenging enzymes and it protected from oxidative stress in stress-resistant mice [70]. Interestingly, we observed that the untreated wild type N2 strain contained less ROS than the knockout mutants. This supports the hypothesis that changes in the neurotransmitter system leads to the organism’s inability to process stress well. This is another hint that ROS levels and depression are interconnected. Remarkably, the ROS levels were reduced in all of the nematode strains investigated by us upon treatment with medicinal plant extracts. Thus, the extracts successfully compensated the deficiencies in the mutants. In addition, our results also implied that the extracts were bioavailable. Interestingly, the antioxidant activity determined as Trolox equivalent and antioxidant activity determined in *C. elegans* correlated with each other only to a limited extent. All of the extracts showed high antioxidant activity in Trolox and H2DCFDA assays. Nevertheless. *Valeria officinalis*, which exhibited the strongest TEAC, showed one of the lowest ROS reductions in the H_2_DCFDA assay. Whereas *Withania somnifera*, which has a lower TEAC, showed a higher ROS lowering effect in the worm model. This may be related to the fact that the substances that are responsible for the antioxidant properties might be not always equally membrane-permeable and, thus, not equally taken up by *C. elegans*. These investigations are supported by other authors. For example, *Withania somnifera* re-balanced derailed ROS levels in a neurodegenerative mouse model [73]. *Campsis grandiflora* protected the brain of mice from ROS and reduced the depressant-like severity [57].

Furthermore, we investigated whether the selected plant extracts increase the resilience to osmotic stress. Hyperosmotic stress leads to protein aggregation and destruction. Additionally, osmotic stress also provokes cellular ROS accumulation [74]. This is quite in accordance with our results demonstrating that the medicinal plants we investigated did not only reduce ROS levels, but also osmotic stress. In the untreated state, all of the mutants examined revealed short reaction times to paralysis than the wild type. Here, gene knockouts also made the nematodes more vulnerable to stress. Treatment with the medical plant extracts significantly increased the times to paralysis, making the worms more resistant to stress.

As a next step, we employed a heat stress assay to examine the effect of the extracts on survival of the different nematodes. Heat stress represents a factor that is responsible for the stimulation of the production of ROS in organisms [75]. Therefore, heat stress was applied to test the effect of extracts on the survival rate of nematodes. As expected, the medicinal plants also increased the resistance to heat. *Campsis grandiflora* showed the strongest increase in heat stress resistance (54.2%), followed by *Houttuynia cordata* (52.5%). *Valeriana officinalis* was also very effective in reducing heat stress in *C. elegans*. These results are consistent with the results from the H_2_DCFDA assay, in which *Houttuynia cordata* was also very effective. *Dan Zhi* (28.8% survival) showed the weakest extracts comparing the overall effect. These results indicate that antioxidant activity corresponds to resilience towards heat stress Additionally, mutants were again more vulnerable towards heat stress as compared to untreated N2 wild type.

Because it is known that the increased production of oxygen radicals also contributes to aging [76], extracts were tested for their effectiveness in longevity. Indeed, *Centella asiatica* for example showed significant increase in survival, which was supported by other research findings, where this plant exhibited anti-aggregation potential on α-synuclein aggregation [77]. The Lifespan was enhanced by all extracts in all nematodes. The evaluation of the overall lifespan extension showed that all extracts exhibited similar lifespan extension (around 130%). Differences in the individual mutants may be due to differential signaling pathway activation. Further studies should be undertaken to investigate the influence of the different constituents, because, to the best of our knowledge, we are the first to examine the effects of *Valeriana officinalis* in *C. elegans*. Another study with dehydrocorybulbine, an ingredient of *Corydalis yanhuso*, showed its binding to both serotonin receptor 7 and dopamine receptors, thus reducing depressive-like behaviour in mice [78].

The chemical fingerprints of the nine extracts were provided by using HPLC-HRMS provided. In general, the frequent occurrence of flavonoids and triterpenoids in the extracts speaks for the antioxidant potential of the extracts [79,80]. Ursolic acid, which was present in most of the extracts, is known for its antioxidant properties [81] and antidepressant effects [82], and it was effective in reducing stress in *Chaenorhabditis elegans* dopamine model [43]. *Withania somnifera* itself is known for its antidepressant activity [83] and improvement of cognitive functions [84]. Withanolide A, which was found in the extract of *Withania somnifera*, is also known for its antioxidant and stress-reducing potential [41,85]. These findings could explain the strong effect of *Withania somnifera* in the different stress assays and lifespan assay. Maslinic acid, which was found in *Campsis grandiflora*, also possesses antioxidant activity [86]. The flavone luteolin acts in a neuroprotective and antioxidant manner [87,88]. Alpha-linolenic acid, a constituent of *Houttuynia cordata*, is also neuroprotective [89]. The constituents wighteone and sophoracoumestan A are not yet sufficiently investigated in literature, whereas bavachinin’s antioxidant activity has been described [90]. Further investigations are needed to elucidate the stress relieving and antioxidative potential of the identified constituents.

The results of our experiments indicate that the beneficial activity of the panel of medicinal plant extracts studied have many readouts. Diverse stress stimuli were compensated by these plants. While it is well-known that medicinal plants and natural products are not acting in a mono-specific manner, but have multiple targets [91], this does not imply that they act non-specifically. The results of the present investigation demonstrated that their antioxidant, ROS-scavenging activity were tightly associated with various anti-stress stimuli (antioxidant, osmotic, and heat stress), finally favoring the longer survival of the animals. Therefore, the antioxidant activity of the plant extracts may be central to explain stress resistance and anti-ageing. The present investigation also revealed associations between antioxidant stress response and neurotransmitter receptors, explaining why the medicinal plants tested may contribute to anti-depressive effects.

## 5. Conclusions

In conclusion, the present study demonstrates that extracts from Acanthopanax senticosus, Campsis grandiflora, Centella asiatica, Corydalis yanhusuo, Dan Zhi, Houttuynia cordata, Psoralea corylifolia, Valeriana officinalis, and Withania somnifera exerted strong antioxidant activity by reducing intracellular ROS and stress in wild type and knockout mutants of C. elegans. Additionally, these extracts were able to extend the lifespan of nematodes. Analyses of extract constituents revealed the presence of the antioxidant and stress ameliorating substances ursolic aicd and withanolide A.

Overall, our results experimentally substantiate the use of these plants in traditional medicine and suggest that these plants are promising candidates for rationale antidepressant phytotherapy. Further studies are warranted to explore the underlying mechanisms and translate the results to clinical trials with patients suffering from depression.

## Figures and Tables

**Table 1:** Overview of species selected for the present study.

Species	Family	Traditional/Common Name	Drug	Traditional Use	Origin
*Acorus tatarinowii* Schott.	Acoraceae	*Shi Chang Pu*	Rhizome	improving intelligence antioxidant anti-aging anti-depression	Traditional Chinese Medicine
*Campsis grandiflora* (Thunb.) K. Schum.	Bignoniaceae	Chinese trumpet vine	Flower	carminative depurative diuretic sleep anxiety	Traditional Chinese Medicine
*Corydalis yanhusuo* W.T.Wang	Papaveraceae	*Yan hu suo*	Rhizome	mild depression mild mental disorders emotional disturbances severe nerve damage	Traditional Chinese Medicine
*Dioscorea polystachya* Turcz.	Dioscoreaceae	*Shan yao*; Chinese yam	Rhizome	emotional instability poor appetite diabetes	Traditional Chinese Medicine
*Acanthopanax senticosus* (Rupr. & Maxim.) Harms	Araliaceae	*Eleutherococcus senticosus*; Siberian Ginseng	Herb	fatigue stress inflammation	Traditional Chinese Medicine
*Gardenia jasminoides* J. Ellis	Rubiaceae	*Danh-danh*; Cape jasmine	Fruit	inflammation nervous disorders	Traditional Chinese Medicine
*Gastrodia elata* Bl.	Orchidaceae	Chì jiàn	Rhizome	depression anxiety memory	Traditional Chinese Medicine
*Houttuynia cordata* Thunb.	Saururaceae	*Yu Xing Cao*; Fish mint	Herb	inflammation stress diabetes	Traditional Indian Medicine/Traditional Chinese Medicine
*Centella asiatica* (L.) Urban	Apiaceae	*Gotu Kola*; Centella	Herb	increase general brain function and memory neuroprotection adaptogen	Traditional Indian Medicine/Traditional Chinese Medicine
*Longan Arillus* Lour.	Sapindaceae	*Long Yan Rou*	Fruit	forgetfulness insomnia palpitations caused by fright	Traditional Chinese Medicine
*Humulus lupulus* L.	Cannabaceae	Hops	Flower	Loss of appetite Sleep disorders and temporary insomnia Mental stress and mood disorders	Traditional European Medicine
*Passiflora incarnata* L.	Passifloraceae	Maypop; Passion Flower	Herb	Sleep disorders and temporary insomnia Mental stress and mood disorders	Traditional European Medicine
*Platycladus orientalis* (L.) Franco	Cupressaceae	*Bai Zi Ren*; Oriental thuja	Seed	tonic sedative tranquillizer	Traditional Chinese Medicine
*Psoralea corylifolia* L.	Fabaceae	*Bu Gu Zhi; Babchi*	Fruit	tonic sedative inflammation depression	Traditional Indian Medicine/Traditional Chinese Medicine
*Valeriana edulis* Nutt. ex Torr. & A.Gray	Caprifoliaceae	Tobacco root	Rhizome herb	insomnia anxiety	Traditional European Medicine
*Valeriana officinalis* L.	Caprifoliaceae	Valerian	Rhizome herb	Sleep disorders and temporary insomnia Mental stress and mood disorders	Traditional European Medicine
*Valeriana wallichii* DC.	Valerianaceae	Indian Valerian	Rhizome herb	nervine and sedative in hysteria sedative in nervous anxiety	Traditional Indian Medicine
*Withania somnifera* (L.) Dunal	Solanaceae	*Ashwagandha*; Winter-cherry	Herb	Insomnia Anxiety liver tonic anti-inflammatory	Traditional Chinese Medicine
*Dan-zhi-xiao-yao-san*		-	Atractylodis macrocephale rhizoma, Bupleuri radix, *Angelicae sinensis*, poria, Glycyrrihizae radix, tree peony bark, *Gardenia jasminoides, Paeonia lactiflora* Pall, mint and roasted ginger	neuropsychological disorders psychoemotional depression dysmenorrhea	Traditional Chinese Medicine

**Table 2:** Overview of mutants used for in vivo experiments. The deficient protein as well as the human orthologue and the proteins’ role in depression are shown.

Mutant	Protein	Human Homologue	Role in Depression
AQ866	Ser-4	5-hydroxytryptamine receptor 1A (HTR1A)	Mutations are connected to depression and anxiety
DA1814	Ser-1	5-hydroxytryptamine receptor 2B (HTR2B)	agonism induces an SSRI-like response
DA2100	Ser-7	5-hydroxytryptamine receptor 7 (HTR7)	5-HT7 antagonism antidepressant-like effects
DA2109	Ser-1/Ser-7	HTR2B/HTR7	See Da1814/DA2100
MT9772	Mod-5	Serotonin transporter (SERT)	gene-environment interaction of SERT and stress
RB665	Dop-1	Dopamine receptor D1 (D1)	Stimulation improves depression symptoms
LX703	Dop-3	Dopamine receptor D2 (D2)	Enhanced dopamine signalling improves symptoms
VC289	Prdx-2	Peroxiredoxin 2 (Prdx2)	protective role in cells; oxidative stress protection
QV225	Skn-1	Nuclear factor, erythroid 2 like 2 (Nrf2)	Lower expressions of Nrf2 correlated to depressive disorder

**Table 3:** Antioxidant activity as determined by the H_2_DCFDA assay. The percentage of stress reduction by the plant extracts in the different mutants (±standard deviation) and the stress reduction mean over all mutants are shown. *** *p* < 0.001, ** *p* < 0.01, and * *p* < 0.05.

Stress Reduction (%)	N2	AQ866	DA1814	Da2100	DA2109	MT9772	RB665	LX703	VC289	QV225	Stress Reduction Mean (%)
*Acanthopanax senticosus*	72.4 ± 0.3 ***	74.1 ± 3.1 ***	73.3 ± 0.7 ***	86.1 ± 0.4 ***	66.4 ± 1.4 ***	36.5 ± 0.8 *	82.4 ± 0.5 ***	76.4 ± 2.6 ***	33.4 ± 3.9 **	59.6 ± 3.0 *	66.8 ± 18.0
*Campsis grandiflora*	72.3 ± 3.2 ***	15.6 ± 18.8 n.s.	48.8 ± 8.0 ***	59.6 ± 6.8 **	58.7 ± 8.3 **	34.1 ± 4.9 ***	43.4 ± 0.9 **	20.2 ± 1.1 **	47.9 ± 3.9 ***	71.1 ± 0.8 ***	47.2 ± 19.4
*Centella asiatica*	79.0 ± 0.4 **	63.9 ± 2.2 ***	63.0 ± 3.5 ***	2.9 ± 7.7 n.s.	51.0 ± 4.9 ***	67.7 ± 1.0 ***	48.3 ± 3.5 **	51.7 ± 4.2 ***	28.3 ± 2.4 **	78.0 ± 1.3 **	53.4 ± 23.3
*Corydalis yanhuso*	59.8 ± 4.3 ***	81.5 ± 0.4 ***	62.2 ± 3.6 ***	82.8 ± 0.6 ***	66.3 ± 4.9 ***	26.7 ± 6.3 *	76.4 ± 5.2 **	75.8 ± 1.2 ***	65.2 ± 17.0 **	69.9 ± 1.5 ***	66.7 ± 16.1
*Dan Zhi*	80.2 ± 2.5 ***	67.6 ± 3.3 **	22.2 ± 11.1 *	79.8 ± 1.4 **	24.2 ± 9.2 *	22.2 ± 6.2 **	35.9 ± 6.2 **	17.0 ± 14.2 n.s.	34.1 ± 12.7 ***	46.6 ± 5.5 **	43.0 ± 24.5
*Houttuynia cordata*	73.5 ± 4.2 ***	69.4 ± 2.0 ***	70.6 ± 3.0 ***	86.1 ± 4.7 ***	61.2 ± 2.4 ***	23.4 ± 13.2 n.s.	74.3 ± 4.4 ***	85.5 ± 2.0 ***	50.0 ± 7.8 ***	66.9 ± 3.1 ***	66.1 ± 18.4
*Psoralea corydalis*	45.4 ± 2.0 ***	72.8 ± 4.9 ***	71.9 ± 1.2 ***	58.9 ± 2.4 ***	29.4 ± 45.8 n.s.	40.1 ± 14.1 *	45.7 ± 19.9 *	56.5 ± 3.5 ***	39.0 ± 3.3 ***	78.5 ± 0.9 **	53.8 ± 16.6
*Valeriana officinalis*	81.4 ± 2.5 ***	48.3 ± 1.8 **	43.2 ± 2.5 ***	64.4 ± 8.2 **	37.1 ± 7.8 **	39. 2± 7.2 **	36.3 ± 2.5 **	10.9 ± 6.2 n.s.	49.9 ± 1.1 ***	70.1 ± 0.6 **	48.1 ± 20.0
*Withania somnifera*	71.4 ± 7.1 ***	77.6 ± 0.8 ***	72.2 ± 3.9 ***	85.9 ± 1.1 ***	65.6 ± 5.0 ***	27.7 ± 4.6 *	77.1 ± 3.7 ***	77.6 ± 1.3 ***	87.8 ± 9.7 **	70.7 ± 8.9 **	71.4 ± 16.8

**Table 4:** Effect of plant extracts on osmotic stress. Lifespan is represented as mean in % with standard deviation and overall lifespan in %. *** *p* < 0.001, ** *p* < 0.01, and * *p* < 0.05.

	Control	*Acanthopanax senticosus*	*Campsis grandiflora*	*Centella asiatica*	*Corydalis yanhusuo*	*Dan Zhi*	*Houttuynia cordata*	*Psoralea corylifolia*	*Valeriana officinalis*	*Withania somnifera*
N2	100 ± 11,5	153.8 ± 7.5 *	284.6 ± 8.1 ***	238.5 ± 6.5 ***	173.1 ± 8.9 **	250.0 ± 6.2 ***	215.4 ± 7.1 ***	196.2 ± 7.8 ***	296.2 ± 5.2 ***	196.2 ± 9.8 **
AQ866	100 ± 9.1	136.4 ± 10.0 *	277.3 ± 8.2 ***	295.5 ± 7.7 ***	159.1 ± 8.6 **	190.9 ± 9.5 **	195.5 ± 11.6 **	250.0 ± 7.3 ***	190.9 ± 9.5 ***	209.1 ± 8.7 ***
DA1814	100 ± 0	228.6 ± 10.4 ***	195.2 ± 9.8 ***	176.2 ± 8.1 ***	123.8 ± 7.7 *	214.3 ± 11.1 ***	257.1 ± 11.1 ***	209.5 ± 9.1***	328.6 ± 5.8 ***	338.1 ± 16.9 ***
DA2100	100 ± 0	109.1 ± 4.2 n.s.	222.7 ± 8.2 ***	268.2 ± 6.8 ***	100.0 ± 9.1 n.s.	363.6 ± 3.8 ***	331.8 ± 5.5 ***	191.3 ± 7.3 ***	209.1 ± 6.5 ***	127.3 ± 25.0 n.s.
DA2109	100 ± 8.7	156.5 ± 5.6 ***	169.6 ± 7.7 ***	195.7 ± 8.9 ***	152.2 ± 22.9 n.s.	169.6 ± 10.3 **	173.9 ± 7.5 ***	166.7 ± 11.4 ***	243.5 ± 10.7 ***	191.3 ± 18.2 *
MT9772	100 ± 8.3	91.7 ± 9.1 n.s.	220.8 ± 9.4 ***	162.5 ± 5.1 **	133.3 ± 15.6 n.s.	212.5 ± 7.8***	175.0 ± 11.9 **	235.0 ± 12.5 *	191.7 ± 10.9 **	290.0 ± 8.7 **
RB665	100 ± 0	395.0 ± 11.4 ***	210.0 ± 9.5 ***	310.0 ± 8.1 ***	220.0 ± 9.1 ***	200.0 ± 10.0 ***	320.0 ± 6.3 ***	228.6 ± 8.5 ***	250.0 ± 8.0 ***	252.4 ± 24.1 **
LX703	100 ± 4.8	223.8 ± 10.6 ***	228.6 ± 8.3 ***	238.1 ± 8.0 ***	200.0 ± 11.9 ***	271.4 ± 7.0 ***	195.2 ± 7.3 ***	147.8 ± 6.3 ***	233.3 ± 4.1 ***	139.1 ± 37.7 *
VC289	100 ± 8.7	113.0 ± 7.7 n.s.	173.9 ± 10.0 *	156.5 ± 8.3 *	169.6 ± 7.7 **	152.2 ± 8.6 *	152.2 ± 11.4 *	190.0 ± 8.8 *	204.3 ± 10.6 **	170.0 ± 15.6 n.s.
QV225	100 ± 0	195.0 ± 17.9 ***	235.0 ± 8.5 ***	190.0 ± 10.5 ***	110.0 ± 13.6 n.s.	210.0 ± 11.9 ***	220.0 ± 9.1 ***	206.5 ± 15.8 ***	180.0 ± 13.9***	210.5 ± 8.8 ***
Overall lifespan extension (%)		180.3 ± 88.9	221.8 ± 38.2	223.1 ± 55.2	154.1 ± 38.4	223.4 ± 60.2	223.6 ± 61.2	206.5 ± 34.5	232.8 ± 48.5	210.5 ± 65.8

**Table 5:** Effect of plant extract on lifespan. Lifespan is represented as mean in days with standard deviation. The overall lifespan extension is represented in %. *** *p* < 0.001, ** *p* < 0.01, and * *p* < 0.05.

	Control	*Acanthopanax senticosus*	*Campsis grandiflora*	*Centella asiatica*	*Corydalis yanhusuo*	*Dan Zhi*	*Houttuynia cordata*	*Psoralea corylifolia*	*Valeriana officinalis*	*Withania somnifera*
**N2**	100.0 ± 1.02	113.7 ± 0.89 ***	117.7 ± 3.02 ***	108.1 ± 2.35 ***	114.7 ± 1.77 ***	117.8± 2.59 ***	116.2 ± 1.31 ***	108.6 ± 0.47 ***	106.1 ± 2.87 **	103.0 ± 0.49 **
**AQ866**	100.0 ± 1.33	153.0 ± 0.87 ***	125.3 ± 3.19 ***	155.3 ± 2.58 ***	150.6 ± 0.44 ***	98.8 ± 5.70 ***	151.3 ± 0.88 ***	144.0 ± 0.93 ***	141.3 ± 3.77 ***	152.0 ± 0.44 ***
**DA1814**	100.0 ± 1.18	137.6 ± 1.28 ***	129.4 ± 1.82 ***	126.5 ± 2.79 ***	111.8 ± 2.12 **	131.8 ± 2.05 ***	128.8 ± 0.91 ***	135.9 ± 0.43 *	138.8 ± 1.69 ***	137.0 ± 1.29 ***
**DA2100**	100.0 ± 1.23	130.2 ± 0.95 **	125.5 ± 1.46 **	130.2 ± 1.42 ***	129.6 ± 1.90 ***	137.7 ± 1.35 ***	126.5 ± 2.44 ***	128.4 ± 0.96 *	132.0 ± 1.87 ***	129.6 ± 1.43 ***
**DA2109**	100.0 ± 1.84	127.0 ± 1.45 **	128.2 ± 1.91 ***	120.2 ± 2.55 ***	131.3 ± 0.93 *	126.4 ± 1.94 **	128.2 ± 1.44 *	122.0 ± 3.02 *	133.7 ± 1.38 **	130.7 ± 1.41 *
**MT9772**	100.0 ± 2.04	150.3 ± 0.45 ***	149.0 ± 1.37 ***	180.0 ± 1.22 *	148.3 ± 0.92 *	166.0 ± 1.64 ***	149.0 ± 0.46 **	121.8 ± 1.12 ***	152.8 ± 2.01 *	146.9 ± 0.93 *
**RB665**	100.0 ± 1.92	139.1 ± 0.92 ***	157.7 ± 2.03 ***	151.9 ± 3.38 ***	141.6 ± 1.36 ***	148.1 ± 4.33 ***	139.1 ± 0.92 ***	132.1 ± 1.46 ***	135.9 ± 12.73 ***	143.0 ± 0.90 ***
**LX703**	100.0 ± 0.62	134.2 ± 0.93 **	118.0 ± 3.52 ***	114.9 ± 3.24 ***	139.1 ± 0.45 *	128.8 ± 2.49 ***	138.5 ± 0.90 ***	129.2 ± 0.96 **	116.1 ± 3.74 ***	135.4 ± 0.92 ***
**VC289**	100.0 ± 2.21	114.9 ± 1.44 **	112.2 ± 1.48 *	112.7 ± 1.47 *	107.2 ± 1.55 n.s.	107.7 ± 1.03 n.s.	106.1 ± 1.56 *	110.5 ± 1.50 **	108.3 ± 1.53 *	115.5 ± 0.96 *
**QV225**	100.0 ± 3.37	150.6 ± 1.87 ***	146.6 ± 1.92 ***	125.3 ± 2.24 ***	124.2 ± 4.52 ***	122.5 ± 3.21 ***	118.5 ± 4.74 **	106.2 ± 2.65 ***	132.6 ± 3.81 ***	117.4 ± 5.26 **
**Overall lifespan extension (%)**		135.1 ± 14.0	131 ± 15.1	130 ± 22.8	129.8 ± 15.3	128.6 ± 19.3	130.2 ± 14.5	123.9 ± 12.5	129.8 ± 15.0	131.1 ± 15.3

## Data Availability

The data are available by the authors upon reasonable request.

## Supplementary Materials

The following are available online. Supplementary Material Part 1—Plant extracts constituents; Supplementary Material Part 2—Identification Standard Substances.

## Sample Availability

Samples of the compounds are not available from the authors.
