# Supplementary material for: Increased Stress Resistance and Lifespan in Chaenorhabditis elegans Wildtype and Knockout Mutants—Implications for Depression Treatment by Medicinal Herbs
Source: Molecules. 2021 Mar 24;26(7):1827. doi: 10.3390/molecules26071827 (PMC8036369; doi:10.3390/molecules26071827)
Supplement: Supplementary file 1 [file molecules-26-01827-s001.zip › 24.3.molecules-1055523-supplementary.docx]

**Supplementary Material Part 1 – Plant extracts constituents**

***Acanthopanax senticosus***

Negative mode

| **Peak** | **RT** | **Formula** | **Mass** | **m/z** | **Ion** | **Molecule** |
| --- | --- | --- | --- | --- | --- | --- |
| 7 | 7.26 | C20 H18 O4 S | 354.0929 | 353.0854 | (M-H)- | 3-(2-Furyl)-3-[(4-methylphenyl)sulfonyl]-1-phenyl-1-propanone; (2Z)-3-Oxo-2-(2-thienylmethylene)-2,3-dihydro-1-benzofuran-6-yl cyclohexanecarboxylate |
| 9 | 8.04 | C17 H20 O9 | 368.1087 | 367.1014 | (M-H)- | 3-O-feruloyl-D-quinic acid |
| 14 | 9.05 | C29 H24 O7 S | 516.1238 | 515.1162 | (M-H)- | (2-Benzoyl-5-methoxyphenoxy)(phenyl)acetic 4-methylphenyl anhydride, 2-(3,4-Dimethoxyphenyl)-6,8-dimethyl-4-oxo-4H-chromen-3-yl 2-naphthalenesulfonate, Phenyl 2-(bis{4-[(1-hydroxyvinyl)oxy]phenyl}methyl)benzenesulfonate |
| 20 | 11.22 | C18 H34 O5 | 330.2388 | 329.2315 | (M-H)- | 9,10,18-Trihydroxyoctadec-12-enoic acid |
| 34 | 16.05 | C18 H32 O3 | 296.2338 | 295.2265 | (M-H)- | 2-[(1R,2R,8aS)-2-Hydroxy-2,5,5,8a-tetramethyldecahydro-1-naphthalenyl]ethyl acetate |

Positive mode

| **Peak** | **RT** | **Formula** | **Mass** | **m/z** | **Ion** | **Molecule** |
| --- | --- | --- | --- | --- | --- | --- |
| 8 | 8.66 | C21 H21 N O6 | 383.1377 | 384.1449 | (M+H)+ | Hydrastine |
| 14 | 9.89 | C20 H17 N O4 | 335.1165 | 336.1237 | (M+H)+ | Zanthoxylumine B |
| 32 | 16.05 | C18 H30 O3 | 294.2196 | 295.227 | (M+H)+ | 17-Hydroxylinolenic acid |
| 52 | 21.3 | C29 H61 N2 O5 |  |  |  | Stearamidopropyl glycerylhydroxypropyldimonium chloride |

***Campsis grandiflora***

Negative mode

| **Peak** | **RT** | **Formula** | **Mass** | **m/z** | **Ion** | **Molecule** |
| --- | --- | --- | --- | --- | --- | --- |
| 13 | 8.63 | C33 H36 O10 S | 624.2022 | 623.1946 | (M-H)- | Methyl 2,3,4,6-tetra-O-acetyl-1-C-{4-methyl-3-[(5-phenyl-2-thienyl)methyl]phenyl}-D-glucopyranoside |
| 20 | 10.59 | C15 H10 O6 | 286.0462 | 285.0389 | (M-H)- | Luteolin |
| 29 | 15.74 | C30 H48 O4 | 472.3529 | 471.3458 | (M-H)- | Maslinic acid |
| 33 | 20.67 | C30 H48 O3 | 456.3579 | 455.3507 | (M-H)- | Ursolic acid |
| 34 | 26.24 | C36 H60 O8 | 620.4257 | 619.4184 | (M-H)- | Riparsaponin |

Positive mode

| **Peak** | **RT** | **Formula** | **Mass** | **m/z** | **Ion** | **Molecule** |
| --- | --- | --- | --- | --- | --- | --- |
| 22 | 11.94 | C18 H28 O3 | 292.2035 | 293.2108 | (M+H)+ | Alpha-licanic acid |
| 41 | 17.47 | C16 H22 O4 | 278.1517 | 302.1441 | (M+Na)+ | Emmotin A |
| 15 | 8.63 | C37 H30 O9 | 618.189 | 642.2382 | (M+Na)+ | Globuxanthone E |

***Centella asiatica***

Negative mode

| **Peak** | **RT** | **Formula** | **Mass** | **m/z** | **Ion** | **Molecule** |
| --- | --- | --- | --- | --- | --- | --- |
| 13 | 9.83 | C30 H26 O7 S | 530.139 | 529.1315 | (M-H)- | Palodesangren A |
| 22 | 12.25 | C30 H48 O6 | 504.3424 | 503.3351 | (M-H)- | Madecassic acid |

Positive mode

| **Peak** | **RT** | **Formula** | **Mass** | **m/z** | **Ion** | **Molecule** |
| --- | --- | --- | --- | --- | --- | --- |
| 13 | 9.78 | C30 H44 O3 | 452.3288 | 453.336 | (M+H)+ | Kulactone |

***Corydalis yanhusuo***

Negative mode

| **Peak** | **RT** | **Formula** | **Mass** | **m/z** | **Ion** | **Molecule** |
| --- | --- | --- | --- | --- | --- | --- |
| 14 | 11.78 | C36 H36 N2 O8 | 624.2437 | 623.2366 | (M-H)- | Hyoscyamide |

Positive mode

| **Peak** | **RT** | **Formula** | **Mass** | **m/z** | **Ion** | **Molecule** |
| --- | --- | --- | --- | --- | --- | --- |
| 7 | 9.13 | C20 H25 N O3 | 327.1835 | 328.1907 | (M+H)+ | Panicudine |
| 16 | 11.35 | C31 H29 N O7 | 527.1951 | 528.2023 | (M+H)+ | Aristophyllide C |

***Dan Zhi***

Negative mode

| **Peak** | **RT** | **Formula** | **Mass** | **m/z** | **Ion** | **Molecule** |
| --- | --- | --- | --- | --- | --- | --- |
| 21 | 12.34 | C27 H26 O7 | 462.1681 | 461.1612 | (M-H)- | Pleionesin C |

Positive mode

| **Peak** | **RT** | **Formula** | **Mass** | **m/z** | **Ion** | **Molecule** |
| --- | --- | --- | --- | --- | --- | --- |
| 39 | 14.82 | C31 H47 N O4 | 497.3491 | 520.3381 | (M+Na)+ | N,O-Diacetylsolasodine |
| 45 | 17.63 | C9 H10 N2 | 146.0839 | 147.0913 | (M+H)+ | Myosmine |

***Houttuynia cordata***

Negative mode

| **Peak** | **RT** | **Formula** | **Mass** | **m/z** | **Ion** | **Molecule** |
| --- | --- | --- | --- | --- | --- | --- |
| 26 | 13.01 | C39 H34 O9 | 646.2221 | 645.2147 | (M-H)- | Mulberrofuran O |
| 40 | 16.09 | C18 H32 O3 | 296.2336 | 295.2262 | (M-H)- | 18-Hydroxylinoleic acid |
| 58 | 23.66 | C20 H40 O3 | 328.296 | 327.2888 | (M-H)- | 2-Hydroxyeicosanoic acid |

Positive mode

| **Peak** | **RT** | **Formula** | **Mass** | **m/z** | **Ion** | **Molecule** |
| --- | --- | --- | --- | --- | --- | --- |
| 3 | 9.1 | C15 H10 O7 | 302.0422 | 303.0495 | (M+H)+ | Quercetin |
| 11 | 16.09 | C18 H30 O2 | 278.2243 | 279.2316 | (M+H)+ | Linolenic Acid |
| 22 | 23.53 | C34 H40 O9 | 592.2682 | 593.2754 | (M+H)+ | Isomoreollic acid |
| 25 | 27.27 | C33 H44 O9 | 584.3018 | 607.291 | (M+Na)+ | Euphornin |

***Psoralea corylifolia***

Negative mode

Positive mode

| **Peak** | **RT** | **Formula** | **Mass** | **m/z** | **Ion** | **Molecule** |
| --- | --- | --- | --- | --- | --- | --- |
| 3 | 9.84 | C20 H20 O6 | 356.1259 | 357.1331 | (M+H)+ | Pluviatolide |
| 7 | 11.38 | C20 H18 O5 | 338.1154 | 339.1226 | (M+H)+ | Wighteone |
| 16 | 13.23 | C20 H18 O4 | 322.1203 | 323.1275 | (M+H)+ | Neobavaisoflavone |
| 25 | 15.6 | C20 H14 O5 | 334.0845 | 335.0914 | (M+H)+ | Sophoracoumestan A |
| 29 | 17.16 | C21 H22 O4 | 339.159 | 338.1518 | (M+H)+ | Bavachinin |

***Valeriana officinalis***

Negative mode

| **Peak** | **RT** | **Formula** | **Mass** | **m/z** | **Ion** | **Molecule** |
| --- | --- | --- | --- | --- | --- | --- |
| 44 | 16.1 | C30 H48 O4 | 472.3526 | 471.3454 | (M-H)- | Ursolic acid |

Positive mode

| **Peak** | **RT** | **Formula** | **Mass** | **m/z** | **Ion** | **Molecule** |
| --- | --- | --- | --- | --- | --- | --- |
| 24 | 8.32 | C15 H25 N O5 | 299.1732 | 300.1805 | (M+H)+ | Echinatine |
| 38 | 12.26 | C15 H20 O2 | 232.1461 | 233.1534 | (M+H)+ | Helenin |
| 44 | 14.18 | C30 H46 O2 | 438.3488 | 439.3561 | (M+H)+ | Momordicinin |
| 57 | 17.66 | C44 H36 N | 578.2843 | 579.2913 | (M+H)+ | 9-Methylphenanthrene |
| 68 | 21.4 | C18 H35 N O | 281.2717 | 282.279 | (M+H)+ | Oleamide |

***Withania Somnifera***

Negative mode

| **Peak** | **RT** | **Formula** | **Mass** | **m/z** | **Ion** | **Molecule** |
| --- | --- | --- | --- | --- | --- | --- |
| 43 | 16.12 | C18 H32 O3 | 296.2334 | 295.2261 | (M-H)- | 18-Hydroxylinoleic acid, Coriolic acid |
| 49 | 22.13 | C18 H32 O2 | 280.2386 | 279.2314 | (M-H)- | Linoleic acid |

Positive mode

| **Peak** | **RT** | **Formula** | **Mass** | **m/z** | **Ion** | **Molecule** |
| --- | --- | --- | --- | --- | --- | --- |
| 16 | 9.88 | C20 H17 N O4 | 335.1148 | 336.1219 | (M+H)+ | Zanthoxylumine B |
| 36 | 21.42 | C18 H35 N O | 281.2715 | 282.2788 | (M+H)+ | Oleamide |
| 38 | 26.28 | C22 H38 N2 O | 346.298 | 347.3052 | (M+H)+ | N-Hexadecylnicotinamide |

**Supplementary Material Part 2- Identification Standard Substances**

**Reference substances**

Ursolic acid C_30_H_48_O_3_

Mass:

Withanolide A C_28_H_38_O_6_

Mass: 470.2659

Withanone C_28_H_38_O_6_

Mass: 470.2661

***Acanthopanax senticosus***

Negative mode

| **RT** | **Formula** | **Mass** | **Ion** | **Substance** |
| --- | --- | --- | --- | --- |
| 20.62 | C_30_H_48_O_3_ | 456.3578 | (M-H)- | Ursolic acid |

***Campsis grandiflora***

Negative mode

| **RT** | **Formula** | **Mass** | **Ion** | **Substance** |
| --- | --- | --- | --- | --- |
| 20.67 | C_30_H_48_O_3_ | 456.3578 | (M-H)- | Ursolic acid |

***Centella asiatica***

Negative mode

| **RT** | **Formula** | **Mass** | **Ion** | **Substance** |
| --- | --- | --- | --- | --- |
| 20.68 | C_30_H_48_O_3_ | 456.3578 | (M-H)- | Ursolic acid |

Positive mode

| **RT** | **Formula** | **Mass** | **Ion** | **Substance** |
| --- | --- | --- | --- | --- |
|  | C_30_H_48_O_3_ | 456.3585 | (M+H)+ | Ursolic acid |
|  | C_28_H_38_O_6_ | 470.2665 | (M+Na)+ | Withanone/Withanolide A |

***Corydalis yanhusuo***

Negative mode

| **RT** | **Formula** | **Mass** | **Ion** | **Substance** |
| --- | --- | --- | --- | --- |
| 20.47 | C_30_H_48_O_3_ | 456.3569 | (M-H)- | Ursolic acid |

***Dan Zhi***

Negative mode

| **RT** | **Formula** | **Mass** | **Ion** | **Substance** |
| --- | --- | --- | --- | --- |
| 20.43 | C_30_H_48_O_3_ | 456.357 | (M-H)- | Ursolic acid |

Positive mode

| **RT** | **Formula** | **Mass** | **Ion** | **Substance** |
| --- | --- | --- | --- | --- |
| 20.68 | C_30_H_48_O_3_ | 457.3652 | (M+H)+ | Ursolic acid |

***Houttuynia cordata***

Negative mode

| **RT** | **Formula** | **Mass** | **Ion** | **Substance** |
| --- | --- | --- | --- | --- |
| 20.49 | C_30_H_48_O_3_ | 456.3574 | (M-H)- | Ursolic acid |

***Valeriana officinalis***

Negative mode

| **RT** | **Formula** | **Mass** | **Ion** | **Substance** |
| --- | --- | --- | --- | --- |
| 20.51 | C_30_H_48_O_3_ | 456.356 | (M-H)- | Ursolic acid |

Positive mode

| **RT** | **Formula** | **Mass** | **Ion** | **Substance** |
| --- | --- | --- | --- | --- |
| 20.67 | C_30_H_48_O_3_ | 456.3603 | (M+NA)+ | Ursolic acid |

***Withania Somnifera***

Negative mode

| **RT** | **Formula** | **Mass** | **Ion** | **Substance** |
| --- | --- | --- | --- | --- |
| 20.52 | C_30_H_48_O_3_ | 456.3572 | (M-H)- | Ursolic acid |

Positive mode

| **RT** | **Formula** | **Mass** | **Ion** | **Substance** |
| --- | --- | --- | --- | --- |
| 11.69 | C_28_H_38_O_6_ | 470.2659 | (M+H)+ | Withanolide A/Withanone |
